# Dissipation Dynamics and Residue of Four Herbicides in Paddy Fields Using HPLC-MS/MS and GC-MS

**DOI:** 10.3390/ijerph16020236

**Published:** 2019-01-15

**Authors:** Qian Yu, Ping Zhang, Yuhan He, Zhifeng Xu, Xiulong He, Yuan Hu, Hongjun Zhang, Lin He

**Affiliations:** 1 Key Laboratory of Entomology and Pest Control Engineering, College of Plant Protection, Southwest University, Chongqing 400715, China; qianyu_swu@163.com (Q.Y.); ping17028@163.com (Y.H.); xzf2018@swu.edu.cn (Z.X.); zpcauz@163.com (X.H.); pingz028@163.com (Y.H.); 2 Academy of Agricultural Sciences, Southwest University, Chongqing 400715, China; 3 Institute for the Control of Agrochemicals, Ministry of Agriculture, Beijing 100125, China; zhanghongjun@agri.gov.cn

**Keywords:** herbicide, dissipation, residue, rice, HPLC-MS/MS, GC-MS

## Abstract

The dissipation dynamics and residue of pyrazosulfuron-ethyl, bensulfuron-methyl, acetochlor, and butachlor in paddy fields at Good Agricultural Practices (GAP) condition were carefully investigated in this study. The four herbicides’ residues were determined based on a quick, easy, cheap, rugged, safe (QuEChERS) method coupled with HPLC-MS/MS and GC-MS. The limit of detection (LOD) for pyrazosulfuron-ethyl, bensulfuron-methyl, acetochlor, and butachlor in all matrices ranged from 0.04–1.0 ng. The limit of quantification (LOQ) of the four herbicides ranged from 0.01–0.1 mg/kg. Moreover, the average recoveries of the four herbicides ranged from 78.9–108% with relative standard deviations (RSDs) less than 15% at three different fortified levels for different matrices. The dissipation results indicated that the average half-lives (t_1/2_) of the four herbicides in soil were in the range of 3.5–17.8 days, and more than 95% of the four herbicides dissipated within 5 days in water. Furthermore, the final residues of the four herbicides were all below the LOQ at harvest time. Such results highlight the dissipation dynamics and residue of the four herbicides in a rice cropping system and contribute to risk assessment as well as scientific guidance on the proper and safe application of herbicides in paddy fields.

## 1. Introduction

Rice (*Oryza sativa* L.) is the most important grain and widely cultivated in China [1,2,3,4]. Thus, the quality and safety of rice are highly related to human health and national strategies. Due to the long growth period of rice, weed problems appear frequently in paddy fields, which compete with rice for space, light, water, and nutrients, leading to a loss of about half of rice production [5,6]. With higher yield and quality requirements for rice, herbicides have been extensively used in paddy fields for weed control. Furthermore, the sales of herbicides have become the largest in the pesticide industry, and the wide use of herbicides in agriculture inevitably leads to higher residues in the environment [7]. Studies indicate that herbicide residues could migrate into soil and water after application, which poses a great threat to the quality and safety of rice, as well as human health [8,9].

**Figure 1:**
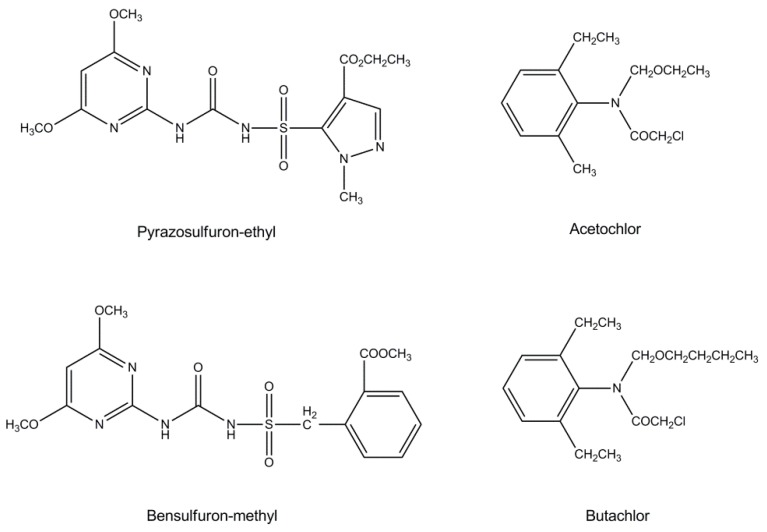
Chemical structures of pyrazosulfuron-ethyl, bensulfuron-methyl, acetochlor, and butachlor.

Pyrazosulfuron-ethyl ethyl 5-[(4,6-dimethoxypyrimidin-2-yl)carbamoylsulfamoyl]-1-methylpyrazole-4-carboxylate, and bensulfuron-methyl methyl 2-[(4,6-dimethoxypyrimidin-2-yl)carbamoylsulfamoylmethyl]benzoate are two commonly used rice herbicides belonging to the sulfonylurea group, which are generally used to control sedges and broadleaf weeds (Figure 1) [10,11]. The action mechanism of sulfonylurea herbicides is to inhibit acetolactate synthase (ALS) activity and block the biosynthesis of branched-chain amino acids of weeds [12]. Acetochlor 2-chloro-N-(ethoxymethyl)-N-(2-ethyl-6-methylphenyl)acetamide and butachlor N-(butoxymethyl)-2-chloro-N-(2,6-diethylphenyl)acetamide are two chloroacetanilide herbicides generally used in rice fields for pre-emergent control of annual grasses and broadleaf weeds [13,14]. The four herbicides inhibit the growth of young shoots and roots, stimulate root-like deformities, and lead to the death of weeds after application in rice cropping systems [15,16,17,18]. Although the half lethal doses(LD_50_) of four herbicides in rats are all over 5000 mg/kg according to the Pesticide Manual [19], the four herbicides are extensively used in paddy fields in consideration of good selectivity, high efficiency, and low toxicity. Previous studies primarily concentrated on herbicidal activity and residue levels of four herbicides in rice cropping systems [10,20,21]. The results indicated that pyrazosulfuron-ethyl, bensulfuron-methyl, acetochlor and butachlor were safe and the residues in rice were lower than the maximum residue limit (MRL) at the recommended dosage [19,20]. However, weed resistance to these four herbicides increased gradually due to widespread use in paddy fields, which inevitably increased the dosage of these four paddy herbicides [22,23,24,25]. Such phenomenon constitutes a serious threat to cropping ecosystem assessment and herbicide residues in rice.

Considering the complexity of matrices in paddy fields, a sensitive, rapid, and reliable sample preparation method is necessary, which should facilitate extraction, enhance enrichment of the target compound, and reduce interferences as much as possible. Solid-phase extraction, supercritical-fluid extraction, liquid-liquid extraction and QuEChERS are generally adopted in paddy sample preparation, and QuEChERS is the most frequently used method [26]. Analytical methods for pyrazosulfuron-ethyl and bensulfuron-methyl have been mainly focused on high performance liquid chromatography (HPLC) [27], capillary electrophoresis (CE) [28], gas chromatography-tandem mass spectrometry (GC-MS) [29], immunoassay [30], and liquid chromatography-tandem mass spectrometry (LC-MS) [31]. Acetochlor and butachlor analysis have been primarily performed on GC [32], HPLC [21], and GC-MS [33]. HPLC-MS/MS and GC-MS have higher sensitivity and precision and lower detection limits than traditional methods at trace levels in various matrices [34,35]. In addition, the combination of QuEChERS and mass spectrometry has been considered as the most sensitive, rapid, and reliable method for pesticide residue analysis in different matrices. 

In this study, a simple and reliable QuEChERS method coupled with HPLC-MS/MS and GC-MS methods was established to determine pyrazosulfuron-ethyl, bensulfuron-methyl, acetochlor, and butachlor residues in rice cropping systems. The dissipation dynamics of the four herbicides in water and soil, as well as the final residues in rice hull and husked rice, were carefully investigated under Good Agricultural Practices (GAP) use. Such results highlight the dissipation dynamics and residue of the four herbicides in rice cropping systems and contribute to risk assessment of herbicide residues in rice, as well as providing scientific guidance on the proper and safe application of rice herbicides in paddy fields.

## 2. Materials and Methods 

### 2.1. Chemicals and Equipment

Pyrazosulfuron-ethyl (purity = 95%), bensulfuron-methyl (purity = 97%), acetochlor (purity = 92%), and butachlor (purity = 92.5%) were obtained from the Institute for Control of Agrochemicals (Beijing, China). Pyrazosulfuron-ethyl WP (10%), bensulfuron-methyl WP (10%), acetochlor WP (10%), and butachlor EC (900 g/L) were purchased from commercial sources. Acetonitrile, methanol, and formic acid were HPLC grade and purchased from Thermo Fisher Scientific (Waltham, MA, USA). Ethyl acetate, acetone, sodium chloride, and anhydrous magnesium sulfate were analytical grade and bought from J&K Scientific Co., Ltd. (Beijing, China). Graphitized carbon black (GCB) and primary secondary amine (PSA) were purchased from Agela Technologies (Tianjing, China). Ultra-pure water was generated using a Milli-Q purification system from Millipore (USA). Pyrazosulfuron-ethyl, bensulfuron-methyl, acetochlor, and butachlor stock standard solutions were prepared with corresponding organic solvent and stored at −20 °C.

Pyrazosulfuron-ethyl and bensulfuron-methyl were analyzed on an Agilent 6410 high performance liquid chromatography-tandem triple quadrupole mass spectrometry equipped with electrospray ionization source (Agilent Technologies, Santa Clara, CA, USA). An Agilent 7890-5977B gas chromatography-tandem mass spectrometry (Agilent Technologies, USA) was used to determine acetochlor and butachlor. A Sigma 3K15 microcentrifuge (St. Louis, MO, USA), Xiangyi L550 centrifuge (Hunan, China), ME204 analytical balance (Sartorius, Germany), IKA T18 grinder (IKA, Germany), and ZWFR-200 shaker (Zhicheng, China) were adopted in sample preparation.

### 2.2. Field Experiment Design 

Field experiments including the degradation dynamics and final residues in supervised field trials were conducted in Chongqing municipality in 2017 (Table 1). All the experiments were designed based on the “Guidelines on pesticide residue trials (NY/T 788-2004)” published by the Institute for the Control of Agrochemicals, Ministry of Agriculture and Rural Affairs of the People’s Republic of China [36]. The area of the field experiment plot was 30 m^2^ and each treatment had three replicated plots. Furthermore, a buffer area was designed to isolate the experiment plots. 

The degradation dynamics experiments were conducted with two dosage levels, pyrazosulfuron-ethyl (22.5 g a.i.ha^−1^, the recommended dosage and 45 g a.i.ha^−1^, double of the recommended dosage), bensulfuron-methyl (26.2 g a.i.ha^−1^, the recommended dosage and 52.4 g a.i.ha^−1^, double of the recommended dosage), acetochlor (52.5 g a.i.ha^−1^, the recommended dosage and 105 g a.i.ha^−1^, double of the recommended dosage) and butachlor (112.4 g a.i.ha^−1^, the recommended dosage and 224.8 g a.i.ha^−1^, double of the recommended dosage), respectively. All the four herbicides were sprayed one time after rice transplanting. Representative 2 kg paddy soil and 500 mL water samples were collected randomly in each plot at 0 (2 h post-treatment), 5, 10, 20, 30, 40, 80 days and pre-harvest interval (PHI) of 7 days after herbicides application. The representative 2 kg rice samples were randomly collected at pre-harvest interval (PHI) of 7 days. All the collected paddy soil, water, and rice samples were stored at −20 °C, respectively.

### 2.3. Analytical Procedure

#### 2.3.1. Sample Preparation

All the samples were thawed at room temperature. 5 g of soil, 5 g of husked rice, 2 g of rice hull and 5 mL of water were weighed into a 50 mL polypropylene centrifuge tube, respectively. 5 mL of purified water with 1% formic acid were added to the rice hull and husked rice sample. 10 mL of acetonitrile was added in all samples for extraction. All the samples were shaken vigorously for 1 min, then 3 g of sodium chloride was added, and samples were oscillated for 30 min in an air bath oscillator at 300 rpm. After that, sample tubes were exposed to ultrasonic vibration for 10 min, and then centrifuged at 3500 rpm for 5 min. 

For soil and water samples, 1 mL of the upper layer was placed into a 2 mL centrifuge tube including 20 mg of PSA and 100 mg of anhydrous magnesium sulfate. The samples were vortexed again for 1 min and then centrifuged at 10,000 rpm for 5 min. The upper extract was filtered through a 0.22 μm filter and transferred into a 2 mL autosampler vial for HPLC-MS/MS or GC-MS analysis, individually. 

For husked rice and rice hull samples, 10 mL of the upper layer was transferred to a 100 mL conical flask, evaporated to dryness at 35 °C on a rotary vacuum evaporator, reconstituted with 1 mL of acetonitrile and transferred into a 2 mL single-use centrifuge tube including 50 mg of PSA, 10 mg of GCB, and 150 mg of anhydrous magnesium sulfate. The sample was vortexed vigorously for 1 min and centrifuged on a microcentrifuge at 10,000 rpm for 5 min. The resulting supernatant was filtered through a 0.22 μm filter and transferred into a 2 mL autosampler vial for HPLC-MS/MS or GC-MS analysis, respectively. 

#### 2.3.2. HPLC-MS/MS Analysis

**Figure 2:**
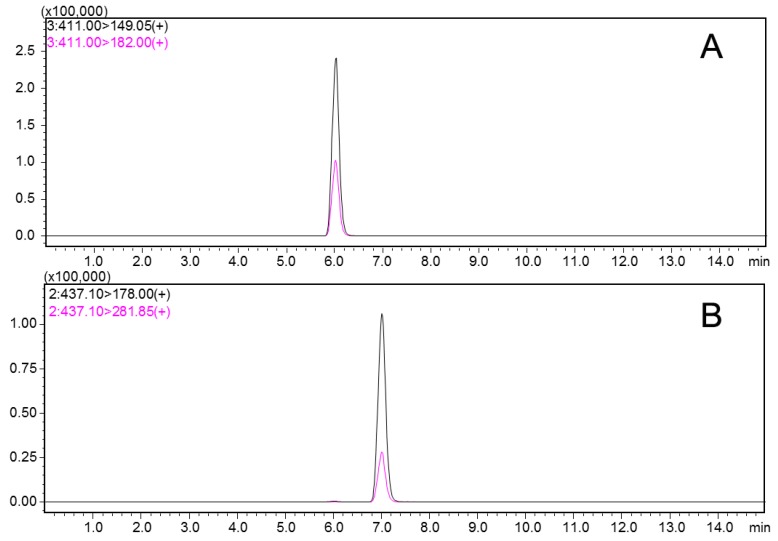
The LC-MS/MS chromatograms of pyrazosulfuron-ethyl (**A**); bensulfuron-methyl (**B**) standards in multiple reaction monitoring (MRM) mode.

The mobile phase was solvent A (methanol) and solvent B (0.1% formic acid in water) (*v/v* = 90:10) with the flow rate of 0.3 mL/min. A sample of 5 μL was injected and the herbicides were separated on an Agilent ZORBAX SB-C_18_ reverse-phase column (50 mm × 2.1 mm, 3 μm). Nitrogen was used as both nebulizer and collision gas in HPLC-MS/MS analysis. The electrospray ionization source (ESI) parameters were as follows: drying gas temperature, 350 °C; gas flow, 8.0 mL/min; nebulizer gas, 35 psi; and capillary voltage, 3000 V. The positive multiple reaction monitoring (MRM) mode was used for monitoring ions transitions. An Agilent Mass Hunter software package was used for method development and data acquisition. Under the above condition, the retention time of pyrazosulfuron-ethyl and bensulfuron-methyl were 7.08 and 6.12 min, individually. The MS parameters and representative chromatograms of pyrazosulfuron-ethyl and bensulfuron-methyl are shown in Table 2 and Figure 2.

#### 2.3.3. GC-MS Analysis

**Figure 3:**
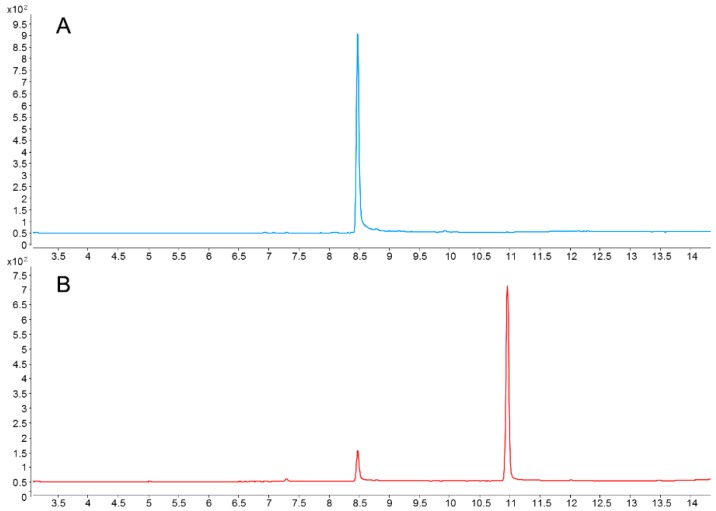
The GC-MS chromatograms of acetochlor (A, m/z = 146) and butachlor (B, m/z = 176) standards in selected ion monitoring (SIM) mode.

Acetochlor and butachlor were analyzed on an Agilent 7890-5977B GC-MS system (Agilent, USA). The injector and detector temperature were set at 260 °C and 280 °C, respectively. Helium was served as the carrier gas at a constant flow rate of 1.0 mL/min and a sample of 2 μL was injected into the GC-MS system. The separations of two herbicides were performed on a HP-5 capillary column (30 m × 0.25 mm inner diameter and 0.25 μm film thickness). Oven temperature program was as follows: the column was held initially at 100 °C for 1 min, then ramped at 20 °C/min to 220 °C, ramped at 1 °C/min to 230 °C, further ramped 20 °C/min to 260 °C, and held at 260 °C for 3 min. The MS parameters were as follows: source temperature of 230 °C, emission current of 35 μA, and energy of −70 eV. The ions transitions were operated in the selective ion monitored (SIM) mode. The retention times of acetochlor and butachlor were 8.58 and 11.22 min, individually. The MS parameters and GC-MS chromatograms of acetochlor and butachlor are listed in Table 3 and Figure 3.

#### 2.3.4. Data Analysis

The degradation dynamics of the four herbicides in paddy fields appeared to follow the first-order kinetic reaction and were calculated according to the following equation: $C_{t}=C_{0}e^{-kt}$, where C_t_ and C_0_ are the concentrations of herbicides at time t and time 0 after spraying (mg/kg), respectively, and k is the degradation rate constant [37,38]. The half-life (t_1/2_) of each herbicide was calculated using the equation: $t_{1/2}=\ln2/k$ [39,40].

## 3. Results

### 3.1. Method Validation

The performance of the developed method was validated with linearity, accuracy, precision, limit of detection (LOD), and limit of quantitation (LOQ). In order to obtain realistic and accurate results, linearity was evaluated by using the matrix-matched standard calibrations method to eliminate matrix effects. Excellent linearities were acquired with all the determination coefficients (R^2^) higher than 0.99 in the range of 0.005–0.5 mg/L with five calibration points for pyrazosulfuron-ethyl, bensulfuron-methyl and 0.025–1 mg/L for acetochlor and butachlor, respectively. Quantification was calculated using the calibration curve constructed by linear regressing of herbicide concentrations against peak areas. The accuracy and precision of the method were evaluated by spiking blank samples with corresponding standard solution at three levels (0.01, 0.1, and 0.5 mg/L for pyrazosulfuron-ethyl and bensulfuron-methyl in paddy fields; 0.1, 0.5, and 1 mg/L for acetochlor and butachlor in soil; 0.01, 0.05, and 0.5 mg/L for acetochlor; and 0.05, 0.5, and 1 mg/L for butachlor in husked rice and rice hull; respectively). The average recoveries of pyrazosulfuron-ethyl, bensulfuron-methyl, acetochlor, and butachlor in paddy environments were 81–106.4%, 78.9–102.7%, 87.4–99.5%, and 86.3–108%, respectively, with the relative standard deviation (RSD) below 14.9% (Table 4). The LOD of pyrazosulfuron-ethyl, bensulfuron-methyl, acetochlor, and butachlor in soil, husked rice, and rice hull were 0.04–1 ng, at a signal-to-noise (*S/N*) ratio of 3. The LOQ was 0.01 mg/kg for pyrazosulfuron-ethyl and bensulfuron-methyl in all matrices, 0.01 mg/kg for acetochlor in husked rice and rice hull, 0.05 mg/kg for butachlor in husked rice and rice hull, and 0.1 mg/kg for acetochlor and butachlor in soil, respectively, at a signal-to-noise (*S/N*) ratio of 10. Such results indicated the established methods were qualified in determining the four herbicides’ residue in paddy environments (Table 4).

### 3.2. Dissipation of Four Herbicides in a Rice Field Ecosystem

**Figure 4:**
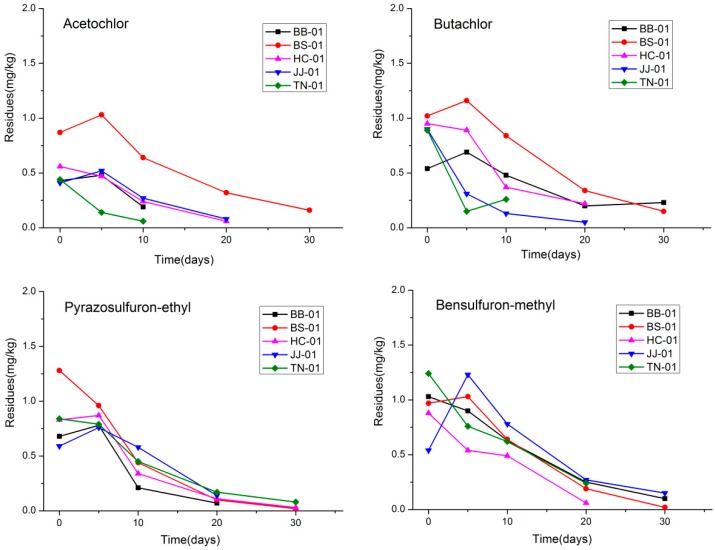
Representative dissipation curves of the four herbicides in soil samples in five different geographic zones after low-dosage application (■, BB-01, ●, BS-01, ▲, HC-01, ▼, JJ-01, ◆, TN-01).

**Figure 5:**
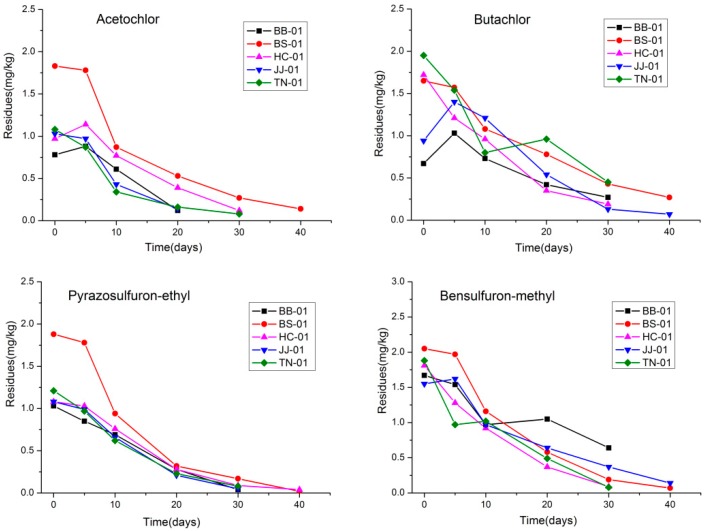
Representative dissipation curves of the four herbicides in soil samples in five different geographic zones after high-dosage application (■, BB-01, ●, BS-01, ▲, HC-01, ▼, JJ-01, ◆, TN-01).

The developed analytical method was applied to dissipation dynamics studies of pyrazosulfuron-ethyl, bensulfuron-methyl, acetochlor, and butachlor after application in an experimental field, respectively. The dissipation curves of four herbicides in soil from different locations are shown in Figure 4 and Figure 5. All the dissipation processes of the four herbicides followed the first-order kinetic reaction, and the dissipation half-life (t_1/2_), as well as other statistical parameters of the four herbicides, are listed in Table 5 and Table 6. The initial residues of pyrazosulfuron-ethyl, bensulfuron-methyl, acetochlor, and butachlor in soil were in the range of 0.4–1.3 mg/kg at low-dosage application and 0.7–2.1 mg/kg at high-dosage application. As expected, the gradual and continuous decreases of the four herbicides in paddy soil were observed at different intervals. The four herbicides degraded fast in soil with the average half-life range from 3–14 days after low-dosage application and 4–20 days after high-dosage application. Furthermore, about 90% of the residues had degraded within 40 days after application. In water samples, the initial residues of pyrazosulfuron-ethyl, bensulfuron-methyl, acetochlor, and butachlor were in the range of 0.18–1.17 mg/kg after low dosage application and 0.34–2.19 mg/kg after high dosage application, individually. The four herbicides degraded fast in water with over 95% of the initial residues dissipated within the first 5 days. Therefore, no dissipation curves and half-lives were obtained in water samples.

### 3.3. Final Residue of Four Herbicides

The final residues of pyrazosulfuron-ethyl, bensulfuron-methyl, acetochlor, and butachlor in husked rice and rice hull samples collected from treated plots at harvest time are shown in Tables S1 and S2. Final residue levels of four herbicides in all samples with different dosage revealed no regional difference was observed at ten experimental sites. The results indicated that the four herbicides’ residues were not detectable or below their respective LOQs in husked rice and rice hull, which indicated pyrazosulfuron-ethyl, bensulfuron-methyl, acetochlor, and butachlor were safe on rice under the recommended dosage and two times the recommended dosage at GAP condition.

## 4. Discussion

### 4.1. Optimization of HPLC-MS/MS Method

The compositions of the mobile phase play a vital role in selectivity, peak shape, and proper retention time in HPLC separations, and formic acid contributed to the protonation of analytes in LC-MS/MS analysis [41]. In this study, 90% methanol and 10% water containing 0.1% formic acid were used as mobile phase for pyrazosulfuron-ethyl and bensulfuron-methyl separation, and there were no interfering peaks near pyrazosulfuron-ethyl and bensulfuron-methyl peaks. Each HPLC-MS/MS run time was less than 10 min. Moreover, the precursor ion and the two most abundant product ions were chosen to construct MRM transitions for pyrazosulfuron-ethyl and bensulfuron-methyl HPLC-MS/MS analysis. In order to obtain powerful sensitivity, the fragmentor voltage and collision energy were optimized for the precursor ion and every specific transition. The suitable HPLC-MS/MS conditions for pyrazosulfuron-ethyl and bensulfuron-methyl are shown in Table 1. In our study, the QuEChERS sample preparation coupled with the HPLC-MS/MS method has a lower LOD, reasonable recovery and relative standard deviation (RSD) than solid phase extraction coupled with liquid chromatography-diode array detector(SPE-LC-DAD) [42] and solid phase extraction coupled with liquid chromatography-tandem mass spectrometry (SPE–LC–MS) [43], respectively (Table S3).

### 4.2. Optimization of GC-MS Method

In this study, acetochlor and butachlor were determined on GC-MS according to their specific product ions and retention time using selected ion monitoring (SIM) mode. The three most abundant ions for acetochlor and butachlor were 146, 162, 174 and 176, 160, and 57, respectively. Thus, 146 and 176 were chosen as quantitation ions for acetochlor and butachlor analysis considering their selectivity and sensitivity in the GC-MS system, and the qualitative ions were 162, 174 and 160, 57 m/z for acetochlor and butachlor, individually. Under the above condition, the retention times of acetochlor and butachlor were 8.58 and 11.22 min, respectively (Table 2). The developed method performed satisfactory results with high sensitivity and specificity in validated experiments and real sample determination. Compared with previous studies, the QuEChERS sample preparation coupled with the GC-MS method developed in our study has a lower LOD, reasonable recovery and relative standard deviation (RSD) than liquid-liquid extraction coupled with gas chromatography-tandem mass spectrometry(LLE-GC-MS) [44], solid phase micro-extraction coupled with gas chromatography-tandem mass spectrometry (SPME–GC–MS) [45], and dispersive liquid phase micro-extraction coupled with gas chromatography-tandem mass spectrometry (DLPME–GC–MS) [33], respectively (Table S4).

### 4.3. Four Herbicides’ Dissipation and Final Residue

In the context of this study, the results indicated that 50% of the initial residues of four herbicides were dissipated in soil within 12 days after treatment. And about 90% of the residues were degraded within 40 days. Furthermore, the dissipations of four herbicides in water were much faster than that in soil, and the herbicides were not detectable after 5 days in water after treatment. Besides the physical and chemical properties of pesticides, other factors such as pH, light, heat, and dissolved oxygen concentration could affect the herbicides’ dissipation in water [10,46]. In soil, studies showed that sulfonylurea herbicides’ dissipation was highly related to pH and acidic soil could accelerate degradation [12,47,48]. Ye et al. reported that soil properties and temperature influenced the dissipation rate of acetochlor in soil [49,50]. Oliveira et al. found that the half-lives of acetochlor in surface soil were 6.51–13.9 days, which was consistent with our study [51]. Rao et al. reported that butachlor dissipation might be due to physical parameters like temperature, wind velocity, and moisture level and the half-lives were 12.5–21.5 days, which is also consistent with our research [14]. The different dissipation trends of the four herbicides in water and soil from different places in our study may also have been affected factors including pH, dissolved oxygen concentration, moisture, soil property, and microorganisms [52,53].

In the final residue trials, no herbicide residue was found in husked rice and rice hull, even though double the recommended dosage of the four herbicides was used on transplanted rice fields. The results indicate the use and consumption of the four herbicides following double the manufacturers’ recommended dosage on rice are safe under an open field environment. Nevertheless, it should be emphasized that the use of herbicides in paddy fields should strictly follow the instructions provided by the manufacture and comply with government regulations.

### 4.4. Dietary Risk Assessment

The acceptable daily intake (ADI) for pyrazosulfuron-ethyl, bensulfuron-methyl, acetochlor, and butachlor are 0.043, 0.2, 0.02 and 0.1 mg/kg (bw), respectively, according to maximum residue limits for pesticides in food in china (GB2763-2016) [54]. In this study, the final residues of four herbicides in the rice samples were all below the LOQs at harvest time. Therefore, the supervised trial median residue (STMR) value may be assumed to be at the LOQ. The LOQ of pyrazosulfuron-ethyl, bensulfuron-methyl, and acetochlor was 0.01 mg/kg, and butachlor was 0.05 mg/kg, respectively. The national estimated daily intake (NEDI) of the four herbicides was defined by the following equation: $\mathrm{NEDI}=\mathrm{STMR}\times \mathrm{Fi}/b_{w}$, where the average body weight ($b_{w}$) of an adult in China was estimated at 60 kg, and the intake of an adult per day (Fi) was 0.3 kg per Chinese person when consuming rice, which was provided based on the dietary guidelines issued by the Health Ministry of China [55]. According to the equation, the NEDI for pyrazosulfuron-ethyl, bensulfuron-methyl, acetochlor, and butachlor was 5 × 10^−5^ mg/kg, 5 × 10^−5^ mg/kg, 5 × 10^−5^ mg/kg, and 2.5 × 10^−4^ mg/kg, respectively. Consequently, the NEDI of the four herbicides is fairly low; the daily dietary intake of pyrazosulfuron-ethyl, bensulfuron-methyl, acetochlor, and butachlor is 0.12%, 0.03%, 0.25%, and 0.25% of the ADI in China, respectively. Such results imply that the potential health risks induced by the four herbicides are not significant in paddy fields, even at double the recommended dosage.

## 5. Conclusions

In the context of this study, a quick, easy, cheap, rugged, safe (QuEChERS) extraction method, coupled with HPLC-MS/MS and GC-MS, was developed to determine the dissipation dynamics and residue of pyrazosulfuron-ethyl, bensulfuron-methyl, acetochlor, and butachlor in rice cropping systems. The average recoveries of the four herbicides ranged from 78.9–108% with relative standard deviations (RSDs) less than 15% at three different fortified levels for soil, rice hull, and husked rice. The dissipation results indicate that the average half-lives of the four herbicides in soil are in the range of 3.5–17.8 days, and more than 95% of all herbicides dissipated within 5 days in water. Furthermore, the final residues of four herbicides were all below LOQ at harvest time. Such results highlight the dissipation dynamics and residue of four herbicides in rice cropping systems and contribute to risk assessment as well as scientific guidance on the proper and safe application of rice herbicides in paddy fields.

## Figures and Tables

**Table 1:** The location of experimental plots.

No.	Experimental Plots	Location
1	BB-01	106.368, 29.747689
2	BB-02	106.389278, 29.881666
3	BS-01	106.11499, 29.534675
4	BS-02	106.159433, 29.613598
5	HC-01	106.397122, 30.125637
6	HC-02	106.181094, 30.185661
7	JJ-01	106.273947, 29.073062
8	JJ-02	106.276326, 29.14402
9	TN-01	105.809623, 30.20763
10	TN-02	105.836951, 30.233446

**Table 2:** Liquid chromatography-tandem mass spectrometry (LC–MS/MS) parameters of pyrazosulfuron-ethyl and bensulfuron-methyl.

Herbicides	Retention Time (min)	Qualifying Ions (m/z)	Quantifying Ions (m/z)	Fragmentor (V)	Collision Energy (V)
Pyrazosulfuron-ethyl	7.08	436.9->178.1	436.9->178.1	135	15
		436.9->281.9			20
Bensulfuron-methyl	6.12	411.0->149.0	411.0->149.0	120	20
		411.0->182.1			20

**Table 3:** Gas chromatography-tandem mass spectrometry (GC-MS) parameters of acetochlor and butachlor.

Herbicides	Retention Time (min)	Qualifying Ions (m/z)	Quantifying Ions (m/z)
Acetochlor	8.58	146.0, 162.0, 174.0	146.0
Butachlor	11.22	176.0, 160.0, 57.0	176.0

**Table 4:** The average recovery, calibration curve, the limits of detection (LODs), and limits of quantification (LOQs) of the four herbicides in soil, husked rice, and rice hull (n = 5).

Herbicides	Sample Matrix	Fortified Level (mg·kg^−^^1^)	Average Recovery (%)	RSD (%)	Calibration Curve	R^2^	LOD (ng)	LOQ (mg·kg^−^^1^)
Pyrazosulfuron-ethyl	Soil	0.01	84.7	1.9	y = 1.11E + 06x − 2358.2	0.9998	0.25	0.01
		0.1	94.5	6.0				
		0.5	91.8	4.2				
	Husked rice	0.01	92.4	3.7	y = 9.31E + 05x + 2447.4	0.9993	0.25	0.01
		0.1	102.8	1.5				
		0.5	102.1	2.6				
	Rice hull	0.01	106.4	3.7	y = 1.00E + 06x − 3245.4	0.9998	0.10	0.01
		0.1	95.3	5.1				
		0.5	81.0	3.6				
Bensulfuron-methyl	Soil	0.01	95.5	10.7	y = 4.63E + 06x + 12105	0.9999	0.25	0.01
		0.05	102.7	5.1				
		0.5	78.9	3.1				
	Husked rice	0.01	83.2	13.4	y = 4.36E + 06x + 11910.5	0.9998	0.25	0.01
		0.05	90.5	14.9				
		0.5	80.0	9.8				
	Rice hull	0.01	94.2	4.6	y = 5.00E + 06x + 9465.6	0.9999	0.10	0.01
		0.05	92.6	9.8				
		0.5	92.4	4.9				
Acetochlor	Soil	0.1	96.3	2.5	y =9.31E + 02x− 5.3453	0.9982	1.0	0.1
		0.5	93.6	5.9				
		1	93.8	0.9				
	Husked rice	0.01	94.6	3.7	y =9.75E + 02x − 6.546	0.9979	0.10	0.01
		0.05	99.5	1.2				
		0.5	97.6	3.8				
	Rice hull	0.01	87.4	9.7	y =9.93E + 02x− 1.5344	0.9994	0.04	0.01
		0.05	90.8	5.8				
		0.5	93.7	7.2				
Butachlor	Soil	0.1	108.0	9.2	y =9.65E + 02x − 1.8882	0.9995	1.0	0.1
		0.5	90.8	6.3				
		1	95.9	2.2				
	Husked rice	0.05	86.3	3.6	y =9.09E + 02x + 7.9348	0.9988	0.50	0.05
		0.5	98.4	3.8				
		1	98.7	3.2				
	Rice hull	0.05	86.9	8.5	y =9.23E + 02+ 3.8587	0.9992	0.20	0.05
		0.5	97.3	9.1				
		1	89.4	5.3				

**Table 5:** The dissipation half-lives of four herbicides in rice soil (low-dosage).

Herbicides	Dosage (g a.i.ha^−^^1^)	Locality	Regression Equation	Determination Coefficient (R^2^)	Half-Life (Days) ^a^
Pyrazosulfuron-ethyl	22.5	BB-01	y = 0.8949e^−0.126x^	0.907	5.5
	22.5	BB-02	y = 0.8806e^−0.129x^	0.976	5.4
	22.5	BS-01	y = 1.6411e^−0.143x^	0.990	4.8
	22.5	BS-02	y = 1.1799e^−0.122x^	0.921	5.7
	22.5	HC-01	y = 1.1129e^−0.118x^	0.974	5.9
	22.5	HC-02	y = 1.0955e^−0.124x^	0.906	5.6
	22.5	JJ-01	y = 0.8676e^−0.078x^	0.759	8.9
	22.5	JJ-02	y = 1.1424e^−0.109x^	0.982	6.4
	22.5	TN-01	y = 1.1852e^−0.113x^	0.954	6.1
	22.5	TN-02	y = 1.0365e^−0.115x^	0.968	6.0
Bensulfuron-methyl	26.2	BB-01	y = 1.2314e^−0.081x^	0.983	8.6
	26.2	BB-02	y = 0.866e^−0.06x^	0.841	11.6
	26.2	BS-01	y = 1.6713e^−0.132x^	0.919	5.3
	26.2	BS-02	y = 1.0613e^−0.1x^	0.852	6.9
	26.2	HC-01	y = 1.1017e^−0.133x^	0.910	5.2
	26.2	HC-02	y = 0.8935e^−0.189x^	0.995	3.7
	26.2	JJ-01	y = 0.9964e^−0.059x^	0.722	11.7
	26.2	JJ-02	y = 1.1328e^−0.097x^	0.931	7.1
	26.2	TN-01	y = 1.2326e^−0.08x^	0.985	8.7
	26.2	TN-02	y = 0.7745e^−0.135x^	0.908	5.1
Acetochlor	52.5	BB-01	y = 0.5111e^−0.082x^	0.651	8.5
	52.5	BB-02	y = 0.731e^−0.112x^	0.966	6.2
	52.5	BS-01	y = 1.112e^−0.062x^	0.948	11.2
	52.5	BS-02	y = 0.7667e^−0.073x^	0.917	9.5
	52.5	HC-01	y = 0.6889e^−0.117x^	0.965	5.9
	52.5	HC-02	y = 0.7935e^−0.051x^	0.867	13.6
	52.5	JJ-01	y = 0.5751e^−0.091x^	0.862	7.6
	52.5	JJ-02	y = 0.7309e^−0.093x^	0.984	7.5
	52.5	TN-01	y = 0.4187e^−0.199x^	0.993	3.5
	52.5	TN-02	y = 0.54e^−0.055x^	0.772	12.6
Butachlor	112.4	BB-01	y = 0.6421e^−0.053x^	0.766	13.1
	112.4	BB-02	y = 1.1605e^−0.07x^	0.953	9.9
	112.4	BS-01	y = 1.3721e^−0.07x^	0.942	9.9
	112.4	BS-02	y = 1.1642e^−0.061x^	0.869	11.4
	112.4	HC-01	y = 1.0269e^−0.08x^	0.918	8.7
	112.4	HC-02	y = 1.5685e^−0.068x^	0.951	10.2
	112.4	JJ-01	y = 0.7114e^−0.141x^	0.960	4.9
	112.4	JJ-02	y = 0.997e^−0.089x^	0.755	7.8
	112.4	TN-01	y = 0.6035e^−0.123x^	0.455	5.6
	112.4	TN-02	y = 1.3817e^−0.074x^	0.964	9.4

^a^ the half-life calculated using the following equation $t_{1/2}=\ln2/k$.

**Table 6:** The dissipation half-lives of the four herbicides in rice soil (high-dosage).

Herbicides	Dosage (g a.i.ha^−^^1^)	Locality	Regression Equation	Determination Coefficient (R^2^)	Half-Life (Days) ^a^
Pyrazosulfuron-ethyl	45	BB-01	y = 1.4646e^−0.106x^	0.915	6.5
	45	BB-02	y = 1.5816e^−0.132x^	0.932	5.3
	45	BS-01	y = 2.6684e^−0.11x^	0.954	6.3
	45	BS-02	y = 1.515e^−0.105x^	0.938	6.6
	45	HC-01	y = 1.4547e^−0.089x^	0.980	7.8
	45	HC-02	y = 1.8413e^−0.118x^	0.875	5.9
	45	JJ-01	y = 1.4889e^−0.106x^	0.963	6.5
	45	JJ-02	y = 1.5209e^−0.082x^	0.951	8.5
	45	TN-01	y = 1.4166e^−0.093x^	0.990	7.5
	45	TN-02	y = 1.4806e^−0.089x^	0.977	7.8
Bensulfuron-methyl	52.4	BB-01	y = 2.2066e^−0.064x^	0.774	10.8
	52.4	BB-02	y = 1.217e^−0.091x^	0.952	7.6
	52.4	BS-01	y = 2.6665e^−0.088x^	0.983	7.9
	52.4	BS-02	y = 1.6514e^−0.09x^	0.962	7.7
	52.4	HC-01	y = 1.1966e^−0.078x^	0.664	8.9
	52.4	HC-02	y = 1.6466e^−0.174x^	0.992	4.0
	52.4	JJ-01	y = 1.8788e^−0.06x^	0.967	11.6
	52.4	JJ-02	y = 1.635e^−0.079x^	0.988	8.8
	52.4	TN-01	y = 2.076e^−0.096x^	0.912	7.2
	52.4	TN-02	y = 0.7745e^−0.075x^	0.653	9.2
Acetochlor	105	BB-01	y = 1.1331e^−0.1x^	0.843	6.9
	105	BB-02	y = 1.7331e^−0.079x^	0.936	8.8
	105	BS-01	y = 1.9839e^−0.066x^	0.985	10.5
	105	BS-02	y = 1.5256e^−0.076x^	0.931	9.1
	105	HC-01	y = 1.3609e^−0.073x^	0.918	9.5
	105	HC-02	y = 1.2829e^−0.055x^	0.866	12.6
	105	JJ-01	y = 1.2586e^−0.107x^	0.954	6.5
	105	JJ-02	y = 1.765e^−0.121x^	0.962	5.7
	105	TN-01	y = 1.0778e^−0.09x^	0.971	7.7
	105	TN-02	y = 1.5539e^−0.115x^	0.896	6.0
Butachlor	224.8	BB-01	y = 0.9324e^−0.039x^	0.796	17.8
	224.8	BB-02	y = 1.5805e^−0.063x^	0.943	11.0
	224.8	BS-01	y = 1.8038e^−0.047x^	0.988	14.7
	224.8	BS-02	y = 1.8759e^−0.052x^	0.919	13.3
	224.8	HC-01	y = 1.7919e^−0.076x^	0.990	9.1
	224.8	HC-02	y = 2.3666e^−0.064x^	0.951	10.8
	224.8	JJ-01	y = 1.7142e^−0.077x^	0.900	9.0
	224.8	JJ-02	y = 1.5782e^−0.079x^	0.938	8.8
	224.8	TN-01	y = 1.7773e^−0.044x^	0.837	15.8
	224.8	TN-02	y = 1.8878e^−0.063x^	0.949	11.0

^a^ the half-life calculated using the following equation $t_{1/2}=\ln2/k$.

## Supplementary Materials

The following are available online at http://www.mdpi.com/1660-4601/16/2/236/s1, Table S1: Final residues of four herbicides in husked rice sample. Table S2: Final residues of four herbicides in rice hull sample. Table S3: Comparison of QuEChERS-HPLC-MS with other analytical methods for determination of sulfonylurea herbicides. Table S4: Comparison of QuEChERS-GC-MS with other analytical methods for determination of amide herbicides.
