# Supplementary material for: Dissipation Dynamics and Residue of Four Herbicides in Paddy Fields Using HPLC-MS/MS and GC-MS
Source: Int J Environ Res Public Health. 2019 Jan 15;16(2):236. doi: 10.3390/ijerph16020236 (PMC6352214; doi:10.3390/ijerph16020236)
Supplement: Supplementary file 1 [file ijerph-16-00236-s001.pdf]

Supplementary Materials

Dissipation Dynamics and Residue of Four Herbicides in Paddy Fields using HPLC-MS/MS and GC-MS

Table S1. Final residues of four herbicides in husked rice sample.

Herbicides	Dosage (g a.i.ha^{-1})	Sprayed Times	Husked Rice Residue (mg·kg^{-1})									
			BB-01	BB-02	BS-01	BS-02	HC-01	HC-02	JJ-01	JJ-02	TN-01	TN-02
Pyrazosulfuron-ethyl	22.5	1	ND	ND	ND	ND	ND	ND	ND	ND	ND	ND
	45	1	ND	ND	ND	ND	ND	ND	ND	ND	ND	ND
Bensulfuron-methyl	26.2	1	ND	ND	ND	ND	ND	ND	ND	ND	ND	ND
	52.4	1	ND	ND	ND	ND	ND	ND	ND	ND	ND	ND
Acetochlor	52.5	1	ND	ND	ND	ND	ND	ND	ND	ND	ND	ND
	105	1	ND	ND	ND	ND	ND	ND	ND	ND	ND	ND
Butachlor	112.4	1	ND	ND	ND	ND	ND	ND	ND	ND	ND	ND
	224.8	1	ND	ND	ND	ND	ND	ND	ND	ND	ND	ND

ND: <0.01 mg/kg (pyrazosulfuron-ethyl, bensulfuron-methyl and acetochlor); ND: <0.05 mg/kg (butachlor).

Table S2. Final residues of four herbicides in rice hull sample.

Herbicides	Dosage (g a.i.ha^{-1})	Sprayed Times	Rice Hull Residue (mg·kg^{-1})									
			BB-01	BB-02	BS-01	BS-02	HC-01	HC-02	JJ-01	JJ-02	TN-01	TN-02
Pyrazosulfuron-ethyl	22.5	1	ND	ND	ND	ND	ND	ND	ND	ND	ND	ND
	45	1	ND	ND	ND	ND	ND	ND	ND	ND	ND	ND
Bensulfuron-methyl	26.2	1	ND	ND	ND	ND	ND	ND	ND	ND	ND	ND
	52.4	1	ND	ND	ND	ND	ND	ND	ND	ND	ND	ND
Acetochlor	52.5	1	ND	ND	ND	ND	ND	ND	ND	ND	ND	ND
	105	1	ND	ND	ND	ND	ND	ND	ND	ND	ND	ND
Butachlor	112.4	1	ND	ND	ND	ND	ND	ND	ND	ND	ND	ND
	224.8	1	ND	ND	ND	ND	ND	ND	ND	ND	ND	ND

ND: <0.01 mg/kg (pyrazosulfuron-ethyl, bensulfuron-methyl and acetochlor); ND: <0.05 mg/kg (butachlor).

Table S3. Comparison of QuEchERS-HPLC-MS with other analytical methods for determination of sulfonylurea herbicides.

Method	Analyte	LOD (ng)	Sample Volume (mL)	Average Recovery (%)	RSD (%)	Ref.
SPE-LC-DAD [a]	pyrazosulfuron-ethyl	300	10	79.2–92.1	9.9–14.8	[1]
	bensulfuron-methyl	300	10	71.4–93.8	7.7–12.9	
SPE–LC–MS [a]	pyrazosulfuron-ethyl	5	500	92.5–98.5	0.4–1.6	[2]
	bensulfuron-methyl	5	500	99.0–104.9	0.6–1.6	
SPE–LC–MS [a]	pyrazosulfuron-ethyl	56–320	5	74.5–114.6	1.4–17.3	[3]
	bensulfuron-methyl	120–630	5	61.1–110.2	2.5–19.4	
QuEchERS -HPLC-MS	pyrazosulfuron-ethyl	0.1–0.25	5	81–102.8	1.5–6.0	This method
	bensulfuron-methyl	0.1–0.25	5	78.9–102.7	3.1–14.9	

[a]:C_{18}.

Table S4. Comparison of QuEchERS-GC-MS with other analytical methods for determination of amide herbicides.

Method	Analyte	LOD (ng)	Sample Volume (mL)	Average Recovery (%)	RSD (%)	Ref.
LLE-GC-MS	acetochlor	2×10^4	10	84.5–93.5	2.20–5.82	[4]
	butachlor	2×10^4	10	66.6–89.5	2.74–7.35	
SPME–GC–MS	acetochlor	1.2	10	90	6.9	[5]
	butachlor	2.7	10	79	8.1	
DLPME–GC–MS	acetochlor	40	5	80.3–108.8	2.9	[6]
	butachlor	3	5	85.2–108.5	1.6	
QuEchERS -GC-MS	acetochlor	0.04–1	5	87.4–99.5	0.9–9.7	This method
	butachlor	0.2–1	5	86.3–108	2.2–9.2	

References

1. Zhou, S.; Hua, X. W.; Wei, W.; Gu, Y. C.; Liu, X.; Chen, J.; Chen, M.; Xie, Y. T.; Zhou, S.; Meng, X. D., Research on Controllable Degradation of Novel Sulfonylurea Herbicides in Acidic and Alkaline Soils. *Journal of Agricultural & Food Chemistry* **2017,** *65*, 7661.
2. Wang, L. B.; Li, C.; Peng, C. F.; Li, X. Q.; Xu, C. L., A Rapid Multi-Residue Determination Method of Herbicides in Grain by GC—MS-SIM. *Journal of Chromatographic Science* **2008,** *46*, 424–429.
3. Xu, X.; Yang, H.; Wang, L.; Han, B.; Wang, X.; Lee, S. C., Analysis of chloroacetanilide herbicides in water samples by solid-phase microextraction coupled with gas chromatography–mass spectrometry. *Analytica Chimica Acta* **2007,** *591*, 87–96.
4. Park, T. S.; Moon, B. C.; Cho, J. R., An Overview of Resistant Weeds to Sulfonylurea Herbicides in Rice Field, Korea. *Korean Journal of Weed Science* **2005,** *25*.
5. Guidelines on pesticide residue trials (NY/T 788-2004). *Institute for the Control of Agrochemicals, Ministry of Agriculture, China* **2004**.
6. Yamada, Y.; Tominaga, T.; Ohsako, T., Microsatellite variability of sulfonylurea-resistant and susceptible populations of Schoenoplectus juncoides (Cyperaceae) in Kinki, Japan. *Weed Research* **2013,** *53*, 429–439.
